# Towards rational engineering of cells: Recombinant gene expression in defined chromosomal loci

**DOI:** 10.1186/1753-6561-5-S8-O6

**Published:** 2011-11-22

**Authors:** Kristina Nehlsen, Leonor da Gama-Norton, Roland Schucht, Hansjörg Hauser, Dagmar Wirth

**Affiliations:** 1 Helmholtz Centre for Infection Research, 38124 Braunschweig, Germany; 2 Instituto de Tecnologia Química e Biológica-Universidade Nova de Lisboa/Instituto de Biológica Experimental e Tecnológica (ITQB-UNL/IBET), P-2781-901 Oeiras, Portugal; 3 InSCREENeX GmbH, 38124 Braunschweig, Germany

## 

The strength of recombinant gene expression is a key property of cell lines for biopharmaceutical protein production. In most stable cell lines the expression vector is stably introduced into the host chromosomal DNA. Apart from the copy number and the used expression control elements the performance of recombinant expression vectors is modulated by genetic and epigenetic features provided by flanking host elements. Since targeted integration is very difficult cell clones with high expression of a recombinant vector are created by random integration and large scale screening for gene expression. This allows the isolation of those rare recombinant cells in which gene expression is optimal. This is usually due to locus-specific influences of the chromosomal surroundings.

We have developed an efficient methodology for targeting expression cassettes to specific chromosomal sites [1,2]]. The method (Flp recombinase mediated cassette exchange - RMCE) allows the repeated use of defined loci by targeting constructs for expression of proteins and viruses, thereby allowing to exploit the positive features of a given integration site [3,4]]. Thereby, a systematic evaluation of the performance of a set of expression vectors in various chromosomal sites becomes feasible.

**Figure 1:**
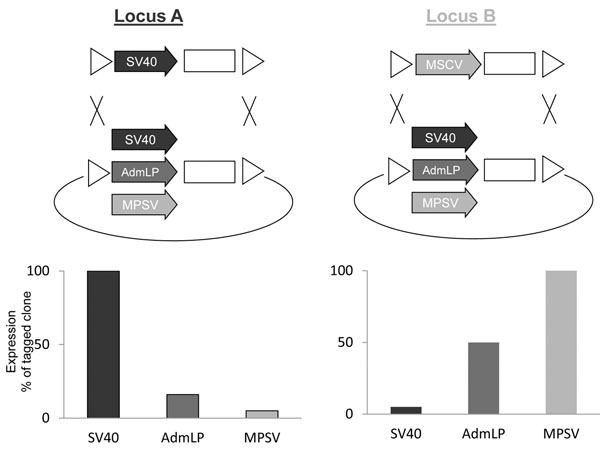
**Mode of tagging defines the optimal targeting cassette.** Two highly potent chromosomal integration sites were screened upon random integration of an expression cassette driven by the SV40 promoter and the MSCV promoter, respectively. By means of Flp recombinase mediated cassette exchange, the screening cassette was exchanged for various expression cassettes, thereby integrating expression cassettes driven by the SV40 promoter, the adenoviral major late promoter (AdmLP) or the MPSV promoter into the same chromosomal site. The expression level upon targeting the various cassettes was determined and related to the expression level of the tagged cell.

In this study we screened for high performance integration sites in HEK293 and CHO-K1 cells supporting expression cassettes driven by a potent promoter. As a read out, production of antibodies and recombinant retroviral vectors were used. Thereby, we could show that high level expression of a given promoter is restricted to defined integration sites, while other sites show only moderate expression (data not shown). An important new finding was that a given chromosomal site is not flexible with respect to the integrated cassette but requires the integration of specific promoters. As illustrated in figure 1, an integration site, identified for supporting high level expression of an SV40 promoter driven cassette fails to adequately support expression of MPSV and adenoviral major late promoter (AdmlP). Vice versa, another site, initially screened for supporting MSCV promoter expression, could restore expression of the highly homologous MPSV promoter while the SV40 promoter and the AdmlP promoter give only moderate expression.

Finally, we tested the impact of the orientation of the cassette in a specific chromosomal site. We found that some integration sites are flexible with respect to the orientation of the expression cassettes while others support expression only in one direction (data not shown).

While classical enhancer elements are known to activate promoters largely independent from the relative position this finding suggests that other cis acting elements affect the incoming cassettes in an orientation dependent manner. Together, this shows that not the nature of integration site and the design of the vector as such define the performance of a producer cell clone. Rather, the interplay between these components defines the level and stability of expression. Since these interactions cannot be predicted, the performance of a vector in a given site has to be evaluated empirically.

In order to exploit favourable sets of chromosomal sites and vectors we made use of bacterial artificial chromosome (BAC) vectors. By recombineering, expression cassettes were integrated into pre-selected chromosomal sites as encoded by BAC vectors. These vectors were randomly integrated into cells by standard transfection protocols. Clones were isolated and evaluated for expression. As expected, highly reproducible expression characteristics were found in individual clones (data not shown). In conclusion, the definition of favorable combinations of specific integration sites and vector design allow the rational exploitation of given chromosomal sites. For this purpose, technologies for site specific genetic manipulation of mammalian cells are essential. This concerns both targeted integration of expression cassettes into defined loci (such as RMCE or site specific nuclease induced homologous recombination) or by transduction of large chromosomal domains (as provided by BAC vectors). These technologies pave the way for predictable and high expression of biotechnologically relevant products such as antibodies and recombinant viral vectors.
